# Role of Epstein-Barr Virus in Breast Cancer: Correlation with Clinical Outcome and Survival Analysis

**DOI:** 10.7150/jca.93631

**Published:** 2024-03-04

**Authors:** Yi-Chiung Hsu, Ming-Han Tsai, Guani Wu, Chien-Liang Liu, Yuan-Ching Chang, Hung-Bun Lam, Pei- Yu Su, Chun-Fan Lung, Po-Sheng Yang

**Affiliations:** 1 Department of Biomedical Sciences and Engineering, National Central University, Taoyuan, Taiwan.; 2 Center for Astronautical Physics and Engineering, National Central University, Taoyuan, Taiwan.; 3 Institute of Microbiology and Immunology, National Yang Ming Chiao Tung University, Hsinchu, Taiwan.; 4 Department of Statistics & Data Science, University of California Los Angeles, Los Angeles, CA, USA.; 5 Department of General Surgery, MacKay Memorial Hospital, Taipei, Taiwan.; 6 Department of Medicine, MacKay Medical College, New Taipei City, Taiwan.

**Keywords:** Epstein-Barr virus, breast cancer, recurrence, survival, Taqman qPCR

## Abstract

**Background:** Breast cancer is the most prevalent cancer among women worldwide. The potential involvement of Epstein-Barr virus (EBV) in breast cancer pathogenesis has been a subject of debate, but its correlation with clinical outcomes remains uncertain.

**Methods:** In this study, we collected 276 pathologically confirmed breast cancer tissue samples from the tissue bank of MacKay Memorial Hospital and the National Health Research Institutes in Taiwan. DNA was extracted from frozen tissue using The QIAamp DNA Mini Kit. The Taqman quantitative PCR method was employed to assess the EBV copy number per cell in these samples, using NAMALWA cells as a reference. We performed statistical analyses, including 2 × 2 contingency tables, Cox regression analysis, and Kaplan-Meier survival curves, to explore the association between clinicopathologic factors and survival outcomes in breast cancer patients. We analyzed both relapse survival, which reflects the period patients remain free from cancer recurrence post-treatment, and overall survival, which encompasses all-cause mortality.

**Results:** Our results revealed a significant association between EBV status and relapse survival (hazard ratio: 2.75, 95% CI: 1.30, 5.86; p = 0.008) in breast cancer patients. However, no significant association was found in overall survival outcomes. Additionally, we observed significant associations between ER status and tumor histologic grade with both overall and relapse survival. Patients with EBV-positive tumors exhibited higher recurrence rates compared to those with EBV-negative tumors. Furthermore, we noted significant correlations between EBV status and HER-2 (p = 0.0005) and histological grade (p = 0.02) in our cohort of breast cancer patients.

**Conclusions:** The presence of EBV in breast cancer tumors appears to exert an impact on patient outcomes, particularly concerning recurrence rates. Our findings highlight the significance of considering EBV status as a potential prognostic marker in breast cancer patients. Nonetheless, further research is essential to elucidate the underlying molecular mechanisms and develop novel therapeutic approaches.

## Introduction

Epstein-Barr virus (EBV), Human papillomaviruses (HPVs), hepatitis viruses HBV and HCV, Kaposi's sarcoma herpesvirus (KSHV), human T-cell leukemia virus (HTLV-I), and Merkel cell polyoma virus (MCPyV) are oncoviruses associated with various cancers^1^. These oncoviruses include various classes of DNA and RNA viruses and induce cancer by a variety of mechanisms, usually after long- term chronic infection. Identification of oncoviruses has provided insights into carcinogenesis and led to interventions such as vaccines, screenings, antivirals, and blood supply checks, aiming to reduce cancer risks. Continued efforts to discover and understand the pathogenesis can drive the development of new preventive and therapeutic strategies.

Epstein-Barr virus (EBV) is belonged to human gammaherpesvirus that infects and establishes life- long infection of the majority of human population. EBV was accidentally discovered in the 1960s during the study of Burkitt's lymphoma, when lymphoma cells were successfully cultured in vitro for the first time^2^. Subsequent examination using electron microscopy confirmed the presence of viral particles known as EBV. EBV has a high prevalence, infecting over 90% of the global population^3^, ^4^. In various Chinese cities, EBV infection is widespread, affecting over 80% of children above 6 years old and 90% of children above 8 years old^5^. The easy transmission of EBV through saliva is a significant factor in its spread. Chinese baby feeding habits, such as mouth feeding or prechewing food for babies, lead to early EBV infections. According to another survey, nearly 98% of Chinese individuals are infected with EBV before reaching 30 years of age^6^. After primary infection, EBV can establish latency in memory B cells, leading to the risk of reactivation during periods of stress, infection, or immunosuppression^7^. The most significant long-term complications associated with EBV infection are the development of malignancies, including multiple types of human lymphomas, nasopharyngeal carcinoma, and gastric carcinoma^8^. Importantly, the malignant cells of found in these EBV-associated malignancies exclusively are infected by EBV whilst the surrounding health tissues are not infected^9^, ^10^, this phenomenon was also reported in breast cancer patients^11^. This phenomenon reveals that EBV infection is critical for the tumorigenesis of the EBV-associated malignancies and the molecular mechanisms how EBV contributes to human cancers are under investigating intensively.

Breast cancer is the first leading cause of cancer death for women that contributes to more than 2 million cancer cases annually^12^. In comparison to nasopharyngeal carcinoma and gastric carcinoma that have been validated with EBV infection, whether EBV contributes to breast cancer is still in controversy. Since the first report in 1995 by Labrecque et al.^13^, research on EBV in breast tumors has mainly focused on detecting and describing the frequency of EBV infected breast tumors. The association between EBV infections with breast cancer had been discussed in many studies but with great variations in between each study due to the different detecting techniques and population^14^-^16^. Studies conducted by various groups have investigated the mechanisms of EBV in breast cancer cells using in vivo and in vitro models. However, the reports on the frequency of EBV infected breast cancer in pathology specimens vary^17^, possibly due to geographical differences and different laboratory detection methods, such as polymerase chain reaction, immunostaining for EBV-encoded nuclear antigen 1/2, or in situ hybridization for small EBV-encoded RNAs. The prevalence of EBV infected breast tumors differs across countries, with lower frequencies observed in the United States, Mexico, and Germany compared to higher frequencies in Argentina, the United Kingdom, France, the Netherlands, Denmark, Algeria, Tunisia, Lebanon, India, and China^17^. Overall, the prevalence of EBV in breast carcinoma worldwide was calculated around 26.37% from the most recently cohort study that included 4,607 breast cancer cases from 26 countries^18^. Another recently published report summarized 24 case control studies that showed the prevalence of EBV was 30.4% of 2,402 breast cancer cases whereas only 7.5% of 1,044 normal and benign breast tissue. (IAC 2021)^19^. One meta-analysis of 30 case- control studies showed that the pooled association between EBV and risk of breast cancer is odds ratio 4.74 (95% CI: 2.92-7.69; Z = 6.30; p < 0.0001) and indicate a strong statistical relationship between EBV infection and risk of breast cancer, suggesting a potential role of EBV infection in the development of breast cancer^18^. Three meta-analyses based on histopathology studies suggest that EBV may increase the risk of breast cancer^18^, ^20^, ^21^. Nevertheless, the correlation between EBV status and clinical outcomes in breast cancer patients remains inconclusive. In this study, our results revealed that EBV play an important role in recurrence of breast cancer patients, especially in Luminal type, Human epidermal growth factor receptor-2 (HER-2) type and higher histological grade (Gr. III) breast cancer patients.

## Materials and methods

### Clinical sample collection

This investigation was performed after approval by the Institutional Review Board of MacKay Memorial Hospital, Taipei, Taiwan (20MMHIS203e) and by the Institutional Review Board of National Biobank Consortium of Taiwan (NBCT No.200066). A total of 276 pathologically confirmed breast cancer samples were obtained from the tissue bank of MacKay Memorial Hospital and National Health Research Institutes in Taiwan.

### DNA Extraction

DNA frozen tissue was extraction using The QIAamp DNA Mini Kit (QIAGEN company, CA). The process involves adding tissue lysis buffer to the frozen tissue, homogenizing the sample, adding proteinase K for protein digestion, transferring the lysate to a spin column with a silica-based membrane for DNA binding, performing wash steps to remove impurities, eluting the purified DNA from the column, quantifying its concentration and purity, and storing it at appropriate temperatures for future use.

### Determination of EBV copy number in the breast cancer samples

The concentration and quality of the genomic DNA extracted from each breast cancer sample were determined by Nanodrop. In case of any chemical residues left during genomic DNA preparation from PFFE samples that might influence the efficacy of qPCR, these genomic DNA were further diluted into 10-, 100-, and 1,000-fold by ultrapure water (thermos) before being subjected to qPCR. To determine the precise EBV copy number per cell, the genomic DNA of NAMALWA cells was extracted and applied as a reference. NAMALWA is an EBV- positive Burkitt's lymphoma cell line with 2 copies of EBV integrated into the genome and is suitable for use as the reference for the evaluation of EBV copy number per cell during qPCR.

We evaluated the copy number of EBV DNA by using the primers and probe specific to BALF5 gene of EBV. The sequence is as follow: BALF5 forward: 5'-CTTTGGCGCGGATCCTC-3'; BALF5 reverse: 5'-AGTCCTTCTTGGCTAGTCTGTTGAC-3'; BALF5 probe: 5'-FAM-CATCAAGAAGCTGCTGGCGGCC-TAMRA-3'.

For all samples, we also applied SYBRgreen qPCR to evaluate the copy number of the ACTIN gene of each sample. The primer sequence: Actin forward: 5'-TGAGCGCGGCTACAGCTT-3' and Actin reverse 5'-TCCTTAATGTCACGCACGATTT-3'. Using NAMALWA BALF5 and actin qPCR results as a reference, the EBV copy number per cell of each breast cancer was able to be quantified^22^.

### Statistical analysis

For each clinical phenotype, we determined the presence of EBV infection in the samples using 2 × 2 contingency tables. To calculate the P-values for the detected mutated genes, we employed Fisher's exact tests. All P-values were considered two-sided, and statistical significance was defined as P < 0.05.

### Survival analysis

We calculated the patients' risk scores from the number of EBV copy and classified them into either positive or negative groups with the medium risk score as the threshold. Kaplan-Meier survival curves were obtained and compared by log-rank tests.

### Cox regression

Cox regression analysis was conducted to evaluate the association between several factors and survival outcomes in breast carcinoma patients. The factors considered in the analysis included EBV, Estrogen receptor (ER), Progesterone receptor (PR), Her-2, TN (triple negative), histological grade, and age. Both standard survival and relapse survival outcomes were analyzed, and for each type of survival, Cox regression analyses were performed. Their results are presented as hazard ratios (HR) with 95% confidence intervals (CI) and corresponding p-values.

## Results

### Classification of the clinical samples and the determination of EBV value

**Figure 1:**
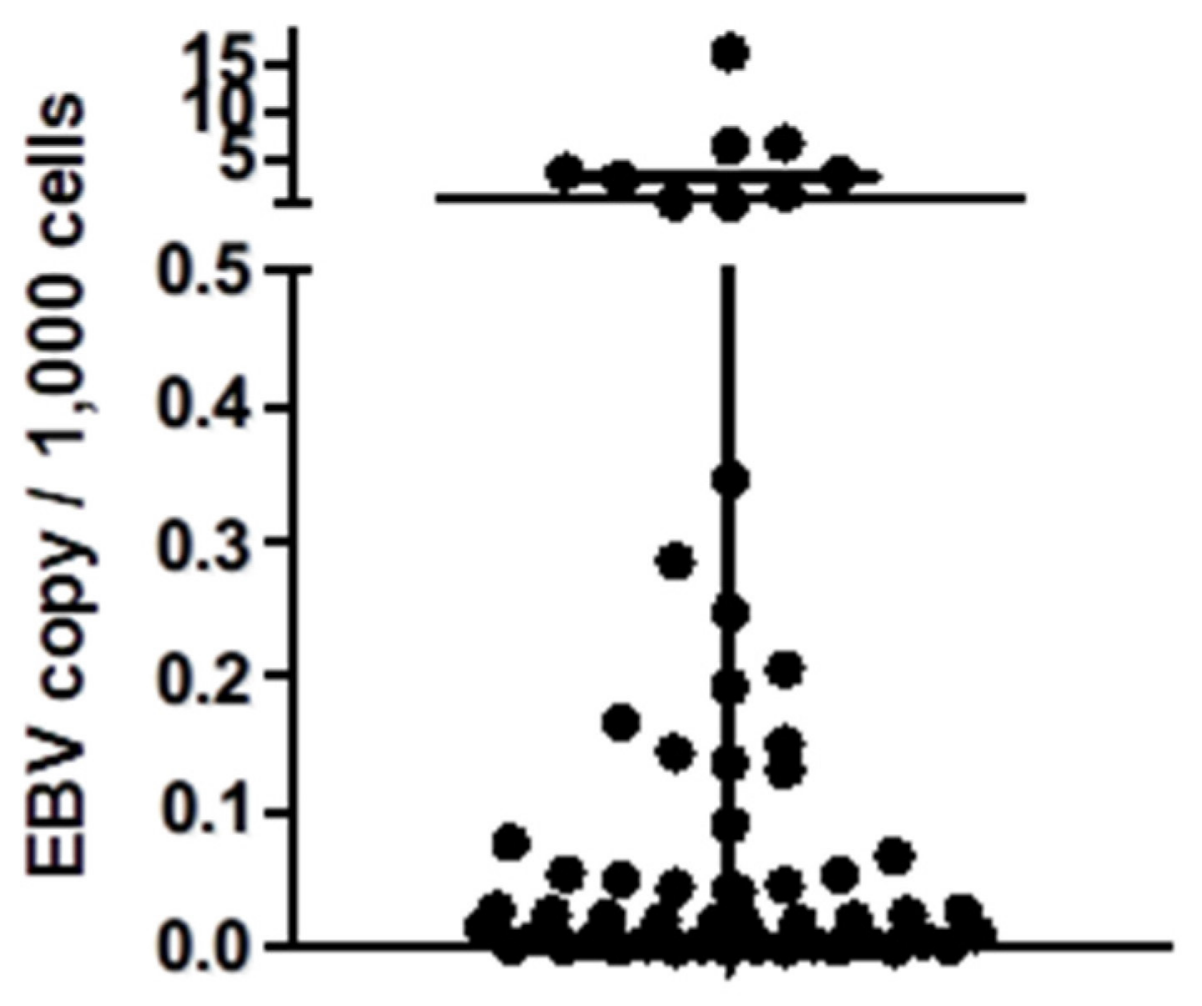
** EBV copy number in the breast cancer samples.** Here we showed the EBV copy number per 1,000 cells of each breast cancer samples in this study. For the samples with negative results from EBV qPCR were not shown.

In this study, we enrolled 276 breast cancer patients, with an average age of 53.8 years (range: 21-86 years). All tumor samples were prepared in frozen tissue and genomic DNA was extracted. We utilized Taqman qPCR to assess the EBV DNA copy number per ug of genomic DNA and calculated the EBV DNA copy per 1,000 cells within the tumor samples (Figure 1). The calculation of copy number per 1,000 total cells is based on the assumption that each cell contains 6 pg of DNA. In our study of 233 samples, we found that the number of EBV genomes was exceptionally low (defined as EBV- negative), measuring less than 0.1 copy per 1,000 total cells^23^. Among these 276 breast cancer samples, 43 were identified as EBV-positive, while 233 were EBV-negative. Pathological features such as ER, PR, Her-2, and tumor histological grade, are shown in Table 1.

The Fisher's exact tests indicated significant associations between EBV status and Her-2 (p = 0.0005) as well as histological grade (p = 0.02) in our breast cancer patients (Table 1). These findings suggest that EBV status is correlated with Her-2 expression and histological grade in breast cancer patients. However, no significant correlations were observed with age, ER, PR, or TN.

### The role of EBV infection in relapse survival of breast cancer patients

**Figure 2:**
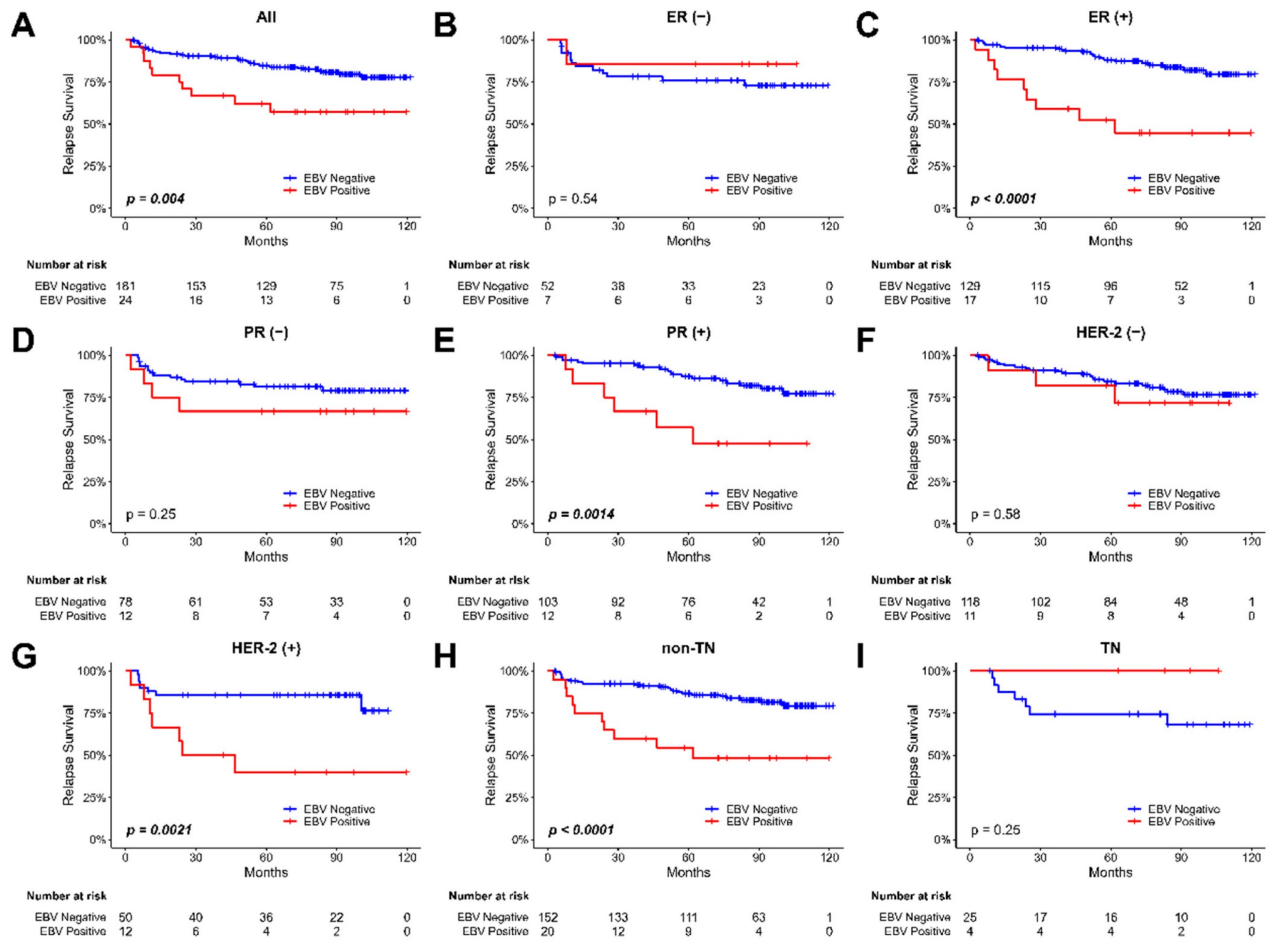
** The Kaplan-Meier plot for the relapse survival of breast cancer patients based on the presence of EBV in tumor samples.** (A) all patients (B) patients with ER-negative tumors, (C) patients with ER-positive tumors, (D) patients with PR-negative tumors, (E) patients with PR-positive tumors, (F) patients with Her-2-negative tumors, (G) patients with Her-2-positive tumors, (H) non-TN (triple-negative) patients, and (I) TN (triple-negative) patients.

We then evaluate the relapse survival of the patients according to the EBV copy value among these tumor samples. The Kaplan-Meier plot for the relapse survival of our breast cancer patients is shown in Figure 2. The 120-month follow-up tracing results showed that there was a significant difference in the recurrence rate of the patients depending on whether the existence of EBV or not in the tumor in all types of breast tumor samples (p = 0.004, Figure 2A). In the analysis of relapse survival, various subgroups, including ER-positive (P<0.0001, Figure 2C), PR-positive (P=0.0014, Figure 2E), Her-2-positive (P=0.0021, Figure 2G), and non-TN tumors (P<0.0001, Figure 2H), all exhibited significant associations with patient outcomes. For assessing the impact of various factors on relapse survival outcomes in our breast cancer patients, we employed Cox regression analysis. Then, we observed a significant association between EBV status and relapse survival in breast cancer patients (HR 2.75; 95% CI, 1.30 to 5.86; p=0.008, shown in Table 2).

### The role of EBV infection in overall survival of breast cancer patients

**Figure 3:**
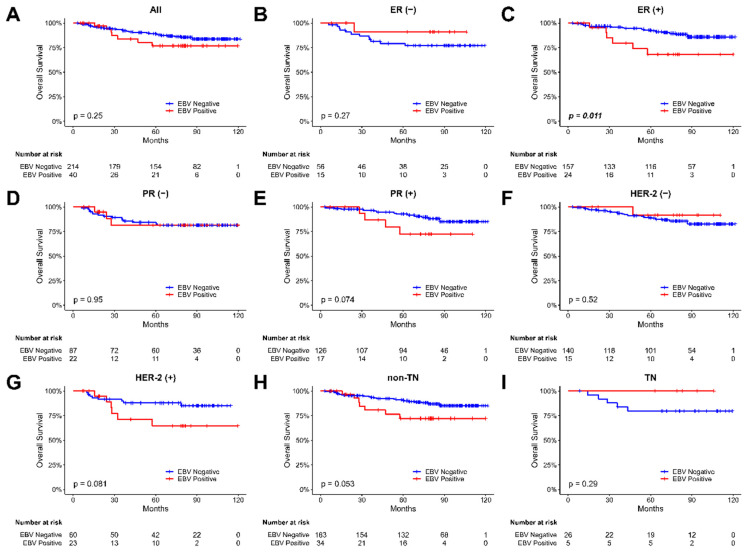
** The Kaplan-Meier plot for the overall survival of breast cancer patients based on the presence of EBV in tumor samples. (A) all patients, (B) patients with ER-negative tumors,** (C) patients with ER-positive tumors, (D) patients with PR-negative tumors, (E) patients with PR- positive tumors, (F) patients with Her-2-negative tumors, (G) patients with Her-2-positive tumors, (H) non-TN (triple-negative) patients, and (I) TN (triple-negative) patients. The abbreviations used are as follows: (-) stands for negative, and (+) stands for positive.

We investigated the correlation between EBV presence and overall survival in breast cancer patients by analyzing overall survival based on the EBV copy value in tumor samples. The Kaplan-Meier plot for the overall survival of our breast cancer patients were shown in Figure 3. During the 120 months follow-up period, our findings revealed a significant difference in overall survival among patients with ER-positive breast tumor samples, depending on whether EBV was present or not (p = 0.011, Figure 3C). However, no significant difference in overall survival rates was observed for patients in the PR and Her-2 groups, irrespective of the EBV status. These results highlight the potential impact of EBV presence on the survival outcomes of specific subgroups of breast cancer patients. However, using Cox regression analysis (HR 1.38; 95% CI, 0.58 to 3.24; p=0.465, Table S1), none of the variables (including EBV status) showed a significant association with overall survival in our breast cancer patients.

### The impact of EBV infection in overall and relapse survival in different histologic grades of breast cancer patients

**Figure 4:**
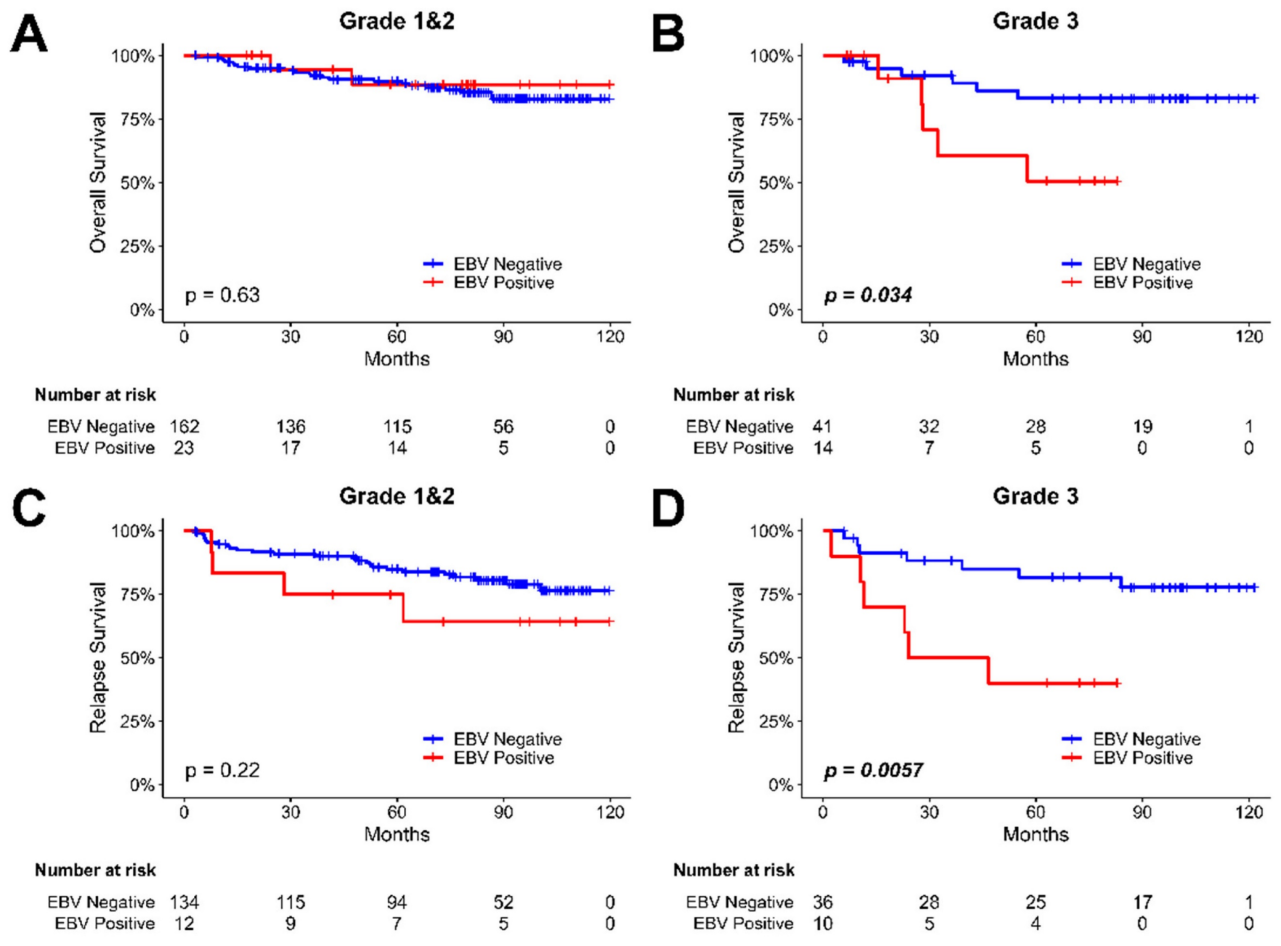
** The Kaplan-Meier plot for the overall and relapse survival of breast cancer patients based on the presence of EBV in tumor samples and tumor histological grade.** (A) the analysis of overall survival in breast cancer patients with tumor grade I & II, focusing on investigating the influence of EBV. (B) the analysis of overall survival in breast cancer patients with tumor histological grade III, focusing on investigating the influence of EBV. (C) the analysis of relapse survival in breast cancer patients with tumor histological grade I & II, focusing on investigating the influence of EBV. (D) the analysis of relapse survival in breast cancer patients with tumor histological grade III, focusing on investigating the influence of EBV.

We examined the relationship between EBV infection and survival outcomes in breast cancer patients, considering variations in tumor histological grade (shown in Figure 4). The results revealed that EBV infection was not significantly associated with survival time in both overall (Figure 4A) and relapse (Figure 4C) survival for patients with grade I and II breast cancer. However, in Gr. III breast cancer patients, a notable difference was observed. The presence of EBV was associated with a shortened overall survival (P=0.034, Figure 4B) and relapse survival (P=0.0057, Figure 4D) compared to EBV- negative patients. EBV play an important role in recurrence of breast cancer patients, especially in Luminal type, Her-2 type and higher histological grade (Gr. III) breast cancer patients.

## Discussion

In this study, we evaluated the presence of Epstein-Barr virus (EBV) in breast cancer samples and its correlation with clinical outcome. Our results showed that there was a significant difference in recurrence rate of patients depending on the presence of EBV in the breast cancer tumor. Breast cancer patients with EBV-positive in tumors had a higher recurrence rate than those with EBV-negative in tumors, as shown in Figure 2. However, there was no significant difference in overall survival rate of patients regardless of the EBV status. These findings suggest that the presence of EBV in breast cancer tumor part may be a useful prognostic marker for relapse survival. Further statistical analyses revealed that breast cancer patients bearing with EBV in their tumor part had higher recurrence rate in the Luminal type, HER-2 type and higher histological grade (Gr. III) breast cancer patients.

EBV infection was correlated with HER-2 status (p=0.0005) in our breast cancer patients (Shown in Table 1). There are studies explored the relationship between Epstein-Barr virus (EBV) and HER-2 in cancer cells. EBV is present in a latent state in infected memory B-cells and EBV-associated cancers. However, cell stimulation or differentiation can induce reactivation of EBV into the lytic replication cycle, where Zta plays a crucial role^24^. This study also assessed the impact of lytic cycle reactivation on changes in gene expression for a panel of Zta-associated cellular genes and revealed substantial conservation in genes associated with Zta-binding sites between epithelial cells and B-cells. Another study investigated the role of Epstein-Barr virus (EBV) immediate-early protein Zta in regulating HER-2 expression and phenotype changes in cancer cells, specifically MDA-MB-453 cells^25^. The results demonstrated that ectopic expression of Zta led to a downregulation of HER-2 protein in various cancer cells. Zta reduced HER-2 mRNA and protein expression in a dose-dependent manner by targeting the HER-2 gene promoter and decreasing its transcriptional activity. These studies provide insights into the relationship between EBV and HER-2, demonstrating that Zta, a key factor in EBV lytic cycle activation, can regulate HER-2 expression and affect the behavior of cancer cells. One study showed that EBV-infected MCF7 and BT474 cells exhibited increased anchorage-independent growth in soft agar^26^. This enhanced colony formation capacity was linked to increased expression and activation of HER2/HER3 signaling cascades. Treatment with HER-2 antibody trastuzumab, a phosphatidylinositol 3-kinase inhibitor, or a MEK inhibitor abolished the tumorigenic capacity of the EBV-infected breast cancer cells, indicating the critical role of HER2/HER3 signaling. These findings may have implications for understanding EBV-associated cancers and potential therapeutic strategies targeting HER-2.

EB virus played some important negative role in relapse survival for Luminal type breast cancer (ER positive or PR positive breast cancer) in our study. There is little study researched the relationship between EBV and the ER for breast cancer but some are explored in the context of nasopharyngeal carcinoma (NPC)^27^. EBV infection in most associated tumors is latent, but the BZLF1 gene product, ZEBRA, can switch the infection from latent to lytic. Recent studies suggest that lytic infection contributes to the oncogenesis of NPC^28^. Additionally, some studies have found correlations between anti-ZEBRA titers and NPC diagnosis and prognosis^27^. According to Dochi's recent study^27^, ZEBRA expression in NPC is associated with increased aromatase (an enzyme that synthesizes estrogen) and ERα expression, as well as worse progression-free survival. In vitro models indicate that ZEBRA expression is induced by estrogen through ERα and contributes to invasion and migration. Notably, this estrogen-ZEBRA axis results in increased viral production without causing cell death, a unique mechanism compared to conventional lytic cells. The authors propose that EBV-infected nasopharyngeal cells may be exposed to high estrogen levels during tumorigenesis due to aromatase expression, leading to the observed effects. This study suggests the importance of active EBV production for NPC development and raises the possibility of using anti-estrogen treatments as therapies for NPC by targeting the estrogen-ERα- ZEBRA axis. ZEBRA's involvement in invasion, migration, and other tumorigenic mechanisms requires further investigation to understand its role fully in NPC prognosis. The estrogen-ERα-ZEBRA axis may explain the possible role of EBV in disease-free survival for Luminal type breast cancer and need further research.

EBV infection was correlated with tumor nuclear grade (p=0.02) (Shown in Table 1) and the prognosis was poor in higher histologic grade breast cancer patients with EBV positive (Figure 3) in our series. This association may be explained by so called “EBVness”. "EBVness" is a human gene expression signature associated with the effects of Epstein-Barr virus (EBV) infection on mammary epithelial cells (MECs)^29^. The virus infects MECs expressing CD21, leading to the expansion of early MEC progenitors, activation of MET signaling, and a differentiation block. When MECs were implanted as xenografts, EBV infection accelerated breast cancer formation in cooperation with activated Ras. EBVness was associated with high-grade, p53 mutation, and poor survival in breast tumors. While EBVness correlated with the presence of EBV DNA in some cases, its presence was not essential for tumor growth, suggesting a "hit and-run" mechanism^30^. Histological tumor grade is a crucial prognostic factor in breast cancer survival, both in general populations and young patients^31^-^33^. It involves visually assessing tissue specimens using hematoxylin and eosin (H&E) stained slides, with the Nottingham modification of the Bloom-Richardson system being the widely used grading method, evaluating nuclear atypia, mitotic rate, and tubule formation. Higher histological grades indicate more aggressive tumors, associated with poorer outcomes, rapid growth, increased metastatic potential, and reduced treatment response. For high grade breast cancer patients, EBV played an important role in carcinogenesis and was a negative disease-free survival factor.

Epstein-Barr virus (EBV) infection has been implicated in influencing treatment responses in cancer patients. For instance, Arbach et al. demonstrated that EBV infection in breast cancer can induce resistance to Paclitaxel (taxol) by upregulating a multidrug resistance gene (MDR1) in vitro-infected MDA-MB-231 cells^34^. This heightened MDR1 expression in paclitaxel-resistant EBV-infected cells (C4A3 and C2G6) likely contributes to the observed resistance. On a positive note, EBV-positive gastric carcinoma has shown improved responses to immune checkpoint therapy^35^, which has yielded lasting clinical remissions in various metastatic cancers. However, the efficacy of immune checkpoint inhibitors (ICIs) as monotherapy for breast cancer, particularly triple-negative breast cancer (TNBC), remains limited^36^. Neoadjuvant chemo-immunotherapy, though, has shown significant benefits in early TNBC patients with positive PD-L1 expression and those in the high-risk subgroup^37^. Our investigation revealed that EBV infection is linked to breast cancer recurrence, particularly in Luminal type, HER-2 type, and higher histological grade (Gr. III) breast cancer patients. Moreover, recent research indicated that EBV-positive breast cancer patients exhibited elevated PD-1/PDL-1 expression compared to their EBV-negative counterparts^11^. Additionally, cases with both EBV infection and PDL1/PD1 expression exhibited the worst disease-free survival and overall survival outcomes. Further studies are warranted to elucidate the potential role of EBV infection in ICI treatment for breast cancer patients, particularly those with non-triple- negative breast cancer, which could lead to the development of novel ICI treatment strategies.

EBV, as a DNA virus, facilitates the detection of viral DNA within cells, irrespective of latency or active infection 1. The examination of many EBV latent proteins poses challenges due to either weak expression or a lack of high-quality antibodies. Additionally, quantifying the expression levels of these proteins through immunohistochemistry is inherently complex. The controversy surrounding the type of EBV latency in breast carcinomas further complicates the selection of suitable latent proteins for our study. Given these challenges, the EBV qPCR method emerged as the optimal choice in our investigation. This method ensures the highest sensitivity for detecting the presence of EBV and provides the most accurate quantification of EBV DNA copy in each examined sample. The limitation of our study, which focused exclusively on active EBV infection in breast cancer tissue samples. The decision to concentrate on active infection was influenced by resource availability and the practicality of the methods employed in our study^38^.

## Supplementary Material

Supplementary table.

## Figures and Tables

**Table 1:** Clinical data statistics based on EBV status. This table shows the association between EBV status and age, ER, PR, HER-2, TN, and histological grade in breast carcinoma patients. The analysis was performed using Fisher's exact test on clinical data from Mackay Memorial Hospital.

Characteristics	N	EBV positive (%)	EBV negative (%)	P-value
All	276	43	233	
*Age (years)*				
<50	113	13 (12)	100 (88)	0.17
≥50	163	30 (18)	133 (82)	
*ER*				
Negative	77	16 (21)	61 (79)	0.17
Positive	197	26 (13)	171 (87)	
*PR*				
Negative	117	23 (20)	94 (80)	0.12
Positive	157	19 (12)	138 (88)	
*HER-2*				
Negative	173	17 (10)	156 (90)	0.0005*
Positive	87	24 (28)	63 (72)	
*TN*				
FALSE	235	37 (16)	198 (84)	1
TRUE	35	5 (14)	30 (86)	
*Histological grade*				
I	35	1 (3)	34 (97)	0.02*
II	162	24 (15)	138 (85)	
III	64	15 (23)	49 (77)	

* means significant p-values (<0.05). Abbreviations: % = percentage of tumors; EBV ¼ Epstein-Barr virus; ER = estrogen receptor; NS = not significant; PR = progesterone receptor; Her-2 = human epidermal growth factor receptor-2; TN = TN, namely ER negative, PR negative, and Her-2 negative.

**Table 2:** Cox regression analysis for relapse survival in breast cancer patients. The table shows the grouping variable, exponentiated hazard ratio coefficient (HR), lower and upper bounds of the 95% confidence interval (CI), and p-value (N=178).

index	HR	95% CI	P-value
EBV positive	2.75	1.30, 5.86	0.008*
ER positive	0.56	0.14, 2.15	0.395
PR positive	1.63	0.56, 4.79	0.373
HER-2 positive	1.18	0.50, 2.76	0.708
TN	0.86	0.19, 3.86	0.839
Grade II	5.40	0.45, 1.72	0.101
Grade III	5.91	0.72, 40.54	0.101
Age >=50	0.88	0.71, 49.39	0.711

* means significant p-values (<0.05).
