# Supplementary material for: Role of Epstein-Barr Virus in Breast Cancer: Correlation with Clinical Outcome and Survival Analysis
Source: J Cancer. 2024 Mar 4;15(8):2403–11. doi: 10.7150/jca.93631 (PMC10937271; doi:10.7150/jca.93631)
Supplement: Supplementary file 1 — Supplementary table. [file jcav15p2403s1.pdf]

Table S1 Cox regression analysis for overall survival in breast carcinoma patients. The table shows the grouping variable, exponentiated hazard ratio coefficient (HR), lower and upper bounds of the 95% confidence interval (CI), and p-value (N=225).

index	HR	95% CI	P-value
EBV+	1.38	0.58, 3.24	0.465
ER+	0.80	0.21, 3.08	0.740
PR+	1.30	0.44, 3.86	0.638
Her-2+	1.12	0.45, 2.78	0.813
TN	0.86	0.18, 4.18	0.849
Grade 2	2.86	0.66, 12.50	0.162
Grade 3	3.90	0.78, 19.54	0.098
Age >=50	1.96	0.89, 4.36	0.097
